# The magic of small-molecule drugs during *ex vivo* expansion in adoptive cell therapy

**DOI:** 10.3389/fimmu.2023.1154566

**Published:** 2023-04-21

**Authors:** Hanwen Zhang, Tenzin Passang, Sruthi Ravindranathan, Ramireddy Bommireddy, Mohammad Raheel Jajja, Lily Yang, Periasamy Selvaraj, Chrystal M. Paulos, Edmund K. Waller

**Affiliations:** ^1^ Department of Hematology and Medical Oncology, Emory University School of Medicine, Atlanta, GA, United States; ^2^ Department of Pathology and Laboratory Medicine, Emory University School of Medicine, Atlanta, GA, United States; ^3^ Winship Cancer Institute, Emory University, Atlanta, GA, United States; ^4^ Departmert of Surgery, University of Alabama at Birmingham Heersink School of Medicine, Birmingham, AL, United States; ^5^ Department of Surgery, Emory University School of Medicine, Atlanta, GA, United States; ^6^ Department of Microbiology and Immunology, Emory University of School of Medicine, Atlanta, GA, United States

**Keywords:** adoptive cell therapy (ACT), chimeric antigen receptors (CAR), *ex vivo* manufacturing, protein kinase inhibitor, PI3K, vasoactive intestinal peptide (VIP), small-molecule drugs, peptide-based drugs

## Abstract

In the past decades, advances in the use of adoptive cellular therapy to treat cancer have led to unprecedented responses in patients with relapsed/refractory or late-stage malignancies. However, cellular exhaustion and senescence limit the efficacy of FDA-approved T-cell therapies in patients with hematologic malignancies and the widespread application of this approach in treating patients with solid tumors. Investigators are addressing the current obstacles by focusing on the manufacturing process of effector T cells, including engineering approaches and *ex vivo* expansion strategies to regulate T-cell differentiation. Here we reviewed the current small-molecule strategies to enhance T-cell expansion, persistence, and functionality during *ex vivo* manufacturing. We further discussed the synergistic benefits of the dual-targeting approaches and proposed novel vasoactive intestinal peptide receptor antagonists (VIPR-ANT) peptides as emerging candidates to enhance cell-based immunotherapy.

## Introduction

Adoptive T-cell therapy (ACT) is a form of cellular immunotherapy in which tumor-reactive T cells recognize and eliminate malignant cells after infusion into patients. Barnes and Loutit initially proposed the ACT concept in 1956, describing the graft-versus-leukemia (GvL) effect of allogeneic hematopoietic stem cell transplantation (HSCT), which represents the earliest clinical example of the adoptive transfer of T cells with anti-cancer activity (1). In the past few decades, cell-based therapies with chimeric antigen receptor (CAR) T cells, engineered T cell receptor (eTCR) T cells, tumor-infiltrating lymphocytes (TILs), and other antigen-specific T cells have rapidly developed and shown enormous clinical potential. CAR T cell therapy, which involves the transfer of allogeneic or autologous T cells modified to express a chimeric antigen receptor (CAR), has gained FDA approval with studies documenting durable remissions in patients with relapsed/refractory (R/R) hematologic malignancies (2–4). However, many patients fail to achieve long-lasting remission due to loss of CAR T cell persistence and functionality (5). Hence, developing methods to counteract T-cell exhaustion and improve functionality is essential to improving ACT efficacy.

T cell exhaustion is a homeostatic mechanism that protects the organism against severe immunopathology from overwhelming CD8 T cell responses (6). Generally, exhausted T (Tex) cells have decreased expression of effector cytokines and increased expression of inhibitory immune checkpoint receptors such as PD-1, TIM-3, LAG-3, TIGHT, and CTLA-4 (7). However, expression of these molecules is also upregulated during early T cell activation, presumably as a homeostatic mechanism that modulates activation downstream of co-stimulatory signaling (8). T cell exhaustion comprises a differentiative process of several stages, accompanied by significant epigenetic reorganization and distinct transcriptional signatures (9, 10). Namely, the expression of TCF1/7, a transcription factor critical to maintaining immunological memory, decreases during the transition from the plastic to the irreversible and fixed dysfunctional chromatin state (11, 12).

Manufacturing of modified T cells is a multi-step process (13). The focus of two main areas of optimizing T-cell therapies are designing optimal genetic modifications of T cells and engineering improved cell activation and culture processes during *ex vivo* T-cell expansion (14). Adding clinically approved compounds, such as monoclonal antibodies and small molecule inhibitors, during the manufacture of cellular products might be a promising strategy to overcome T-cell exhaustion and enhance T-cell cytotoxicity. Adding drugs *ex vivo* represents an alternative to *in vivo* administration as part of a preconditioning regimen or therapy concomitant with T-cell infusion. Preclinical testing of *ex vivo* manufacturing and expansion approaches can identify strategies that yield a more potent adoptive T-cell therapy product with superior anti-tumor activity and persistence after infusion.

Cultures media supplemented with gamma-chain cytokines during *ex vivo* manufacturing, including IL-2, IL-7, IL-15, and IL-21, leads to the expansion of CAR T cells with enhanced proliferation, metabolic profiles, and less terminal differentiation (15). More recently, adding small-molecule compounds targeting tumor cell metabolic signaling pathways has also been explored to enhance T-cell function and persistence. (**Table 1**).

**Table 1:** Summary of small-molecule drugs enhancing adoptive T cells *ex vivo*.

Compound	Names	Target	T Cell Product	Tumor Model	CRS
Protein Kinase Inhibitors					
GDC-0941	Pictilisib	pan-PI3K	gp100-TCR (16)	Melanoma	
LY294002		PI3K α/δ/β	CD33-CAR (17)	AML	
CAL-101	Idelalisib	PI3K δ	gp100-TCR (18) CD19-CAR (19, 20) CD5-CAR (21) Meso-CAR (22)	Melanoma CLL DLBCL Melanoma	
IPI-145	Duvelisib	PI3K δ/γ	gp100-TCR (22) Meso-CAR (22) CD19-CAR (20)	Melanoma CLL	Lower (23)
IPI-549	Eganelisib	PI3K γ	gp100-TCR (22) Meso-CAR (22)	Melanoma	
TGR-1202	Umbralisib	PI3K δ	gp100-TCR (22) Meso-CAR (22)	Melanoma	
bb007		PI3K	BCMA-CAR (24)	MM	
Rapamycin	Sirolimus	mTORC1	EpCAM-CAR (25)	AML	
Akt inhibitor VIII		Akt	TIL (26) gp100-TCR (26) CD19-CAR (27, 28) MiHA-specific (29)	Melanoma Melanoma ALL	
GDC-0068	Ipatasertib	Akt	MiHA-specific (29)		
MK2206		Akt	EpCAM-CAR (30)	Colon Cancer	
Ibrutinib		BTK	CD19-CAR (31–36)	CLL, NHL, ALL	Lower (33)
Acalanrutinib		BTK	CD19-CAR (36)	ALL	
Zanubritinib		BTK	CD19-CAR (37)	B-malignancy	
Dasatinib		TK	CD19-CAR (38)	ALL	
Epigenetic modulators					
Decitabine		DNMT	CD19-CAR (39) CD20-CAR (39) NY-ESO-1-TCR (40)	ALL ALL AML	
Panobinostat		HDAC	Her2-CAR (41) gp100-TCR (41)	PDAC PDAC	
SAHA		HDAC	B7-H3-CAR (42)	TNBC, HNSCC	
Sulforaphane		HDAC	Meso-CAR (43)	Lung Cancer	
JQ1		BRD4	CD19-CAR (44, 45) HLA-A2/MART1_27-35_-TCR (44)	ALL Melanoma	
Immunomodulators					
Lenalidomide			Myeloma-specific (46) CS1-CAR (47) BCMA-CAR (48) CD23-CAR (49) WT1-CAR (50) CD133-CAR (51) HER2-CAR (51) EGFRvIII-CAR (52) CD19-CAR (53)	MM MM MM CLL Wilms Tumor Glioma Breast Cancer GBM DLBCL	
Peptide-based modulators					
VIPhyb		VPAC1/2	CD5 (21) TMV-cultured	DLBCL Colon Cancer	
ANT308		VPAC1/2	T cells		

AML, acute myeloid leukemia; CLL, chronic lymphocytic leukemia; DLBCL, diffuse large B-cell lymphoma; NHL, non-Hodgkin’s lymphoma; PDAC, pancreatic ductal adenocarcinoma; TNBC, triple negative breast cancer; HNSCC, head and neck squamous cell carcinoma; MM, multiple myeloma; GBM, glioblastoma.

## Enhance therapy T-cell *ex vivo* with existing small-molecule drugs

### Protein kinase inhibitors

#### PI3K-AKT-mTOR pathway inhibitors

The most frequent aberrations in cell signaling associated with tumorigenesis, angiogenesis, cell growth, or metastasis are hyperactive PI3K-AKT-mTOR pathways, exemplified by activating mutations of *PIK3CA* and the loss of *PTEN* functionality (54). Hence, the pharmaceutical industry has dedicated significant effort to developing PI3K inhibitors (PI3Ki) as targeted therapies. Mutational activation of PI3K signaling is relatively rare in hematologic malignancies, yet PI3Kδ inhibitors (idelalisib, duvelisib) were approved as a therapy for B-cell malignancies. Aside from suppressing tumor cell growth directly *via* inhibiting intracellular PI3K signaling (55), the beneficial clinical effects of PI3K inhibition in this setting may also be to indirectly activate immune cells with anti-cancer cytotoxicity in the tumor microenvironment. PI3Kγ and PI3Kδ are selectively expressed in leukocytes and are essential in promoting glycolysis and differentiation (56, 57). Indeed, while PI3K inhibitors may dampen many immune cell functions, blocking regulatory T (Treg) cell-mediated suppression of anti-tumor immune responses shows promise in immunotherapy (58). Initially, pan-PI3K inhibitor Pictilisib (GDC-0941) and PI3Kα/δ/β inhibitor LY294002 were found to delay terminal differentiation and preserve a reservoir of memory T cells (T_CM_ and T_EM_) (16, 17). Selective inhibition of PI3Kδ with idelalisib (CAL-101), but not PI3Kα or PI3Kβ, promoted the generation of naïve-like (CD45RA^+^CCR7^+^) and undifferentiated CD8^+^ T cells phenotypes (CCR7^+^CD62L^+^, CD127, Tcf7) that had enhanced proliferative potential, function, and survival (16, 18). Idelalisib also preferentially inhibits human regulatory T-cell function (59). Subsequent studies showed idelalisib-treated T cells, or CAR T cells persisted longer and engrafted better after adoptive transfer into tumor-bearing mice, resulting in improved anti-tumor immunity (18, 19). These cells expressed fewer exhaustion markers (i.e., lower PD1 expression levels) and had a less senescent phenotype (CD27^-^CD28^-^) (21, 60). PI3Kγ, first promoted as a selective immunotherapeutic target in myeloid cells, was later found to be involved in remodeling T-cell differentiation (22, 61, 62). Inhibition of PI3Kγ and PI3Kδ with duvelisib (IPI-145) reprogramed terminal differentiation and the metabolism of CAR T cells to enhance expansion, persistence, and anti-tumor cytotoxicity (20). A recent phase 1 study (NCT03274219) of bb21217, an anti-BCMA CART therapy based on ide-cel that included the PI3K inhibitor bb007 during *ex vivo* culture showed increased enrichment for CD27+/CCR7+ Tm cells, depletion of CD57+ senescent cells, increased CD127 expression, and higher peak *in vivo* CAR T expansion, resulting in improved clinical outcomes in MM patients (24, 63). Excitingly, duvelisib also potently inhibits IL-6 production and cytokine release syndrome (CRS) (23). Two clinical trials were initiated to verify enhanced CAR T-cell functionality (NCT04890236, diffuse large B-cell lymphoma (DLBCL)) and CRS prevention (NCT05044039, non-Hodgkin lymphoma (NHL), acute lymphocytic leukemia (ALL)) in Duvelisib-treated DLBCL patients.

Inhibiting the pathway downstream of PI3K showed a similar effect as direct inhibition of PI3K. mTOR acts intrinsically through the mTORC1 (mTOR complex 1) pathway to regulate memory T-cell differentiation (64). The mTORC1 inhibitor rapamycin promoted memory CD8 T-cell survival, maintenance of a less differentiated phenotype, and improved the functional qualities of CD8 T cells (CD127^High^ CD62L^High^ Bcl2^High^ KLRG1^Low^) (64). Furthermore, rapamycin-pretreated EpCAM CAR T cells had upregulated CXCR4, increased infiltration into the bone marrow, and superior elimination of AML cells in leukemia xenograft mouse models (25). Interestingly, CAR-T cell expansion in IL-15 preserved the stem cell memory (Tscm) phenotype and improved metabolic fitness, likely *via* mTORC1 suppression. However, the inclusion of IL-7 and/or IL-21 in addition to IL15 reduced the beneficial effects of IL-15 on the phenotype and anti-tumor potency of CAR-T (65). Akt functions as a critical signaling node to maintain T cell survival during the effector-to-memory cell transition (66). Like rapamycin, Akt inhibitors, notably Akt-inhibitor VIII and GDC-0068, enhanced the expansion of tumor-specific lymphocytes and promoted the *ex vivo* generation of stem cell memory-like CD8+ T cells (CD62L^high^ CCR7^high^ CXCR4^high^) with a unique metabolic profile and cytokine polyfunctionality (26–29). A pre-clinical study utilizing EpCAM CAR T in a T murine AML model showed that Akt inhibition (MK2206) at the initial stage of CAR T manufacture enhanced the expansion of CAR T cells and CART efficacy *in vivo* (30). Overall, targeting PI3K-AKT-mTOR signaling shows therapeutic potential in improving adoptive T-cell therapy. PI3K inhibition produces a more profound TCF1/7 upregulation than other small molecular TK inhibitors and may elucidate better anti-tumor efficacy *in vivo (* 18 *).*

#### BTK inhibitors (BTKi)

Bruton’s tyrosine kinase (BTK) is a nonreceptor tyrosine kinase initially discovered as a critical component of B cell receptor (BCR) signal transduction in both healthy and malignant B lymphocytes (67, 68). The clinical role of BTK extends beyond its effects on normal and malignant B cells. PI3Kγ can activate BTK to promote phospholipase C (PLC) γ-dependent signaling in hematopoietic cells, including myeloid cells (69). A recent study suggested a regulatory role for BTK in T-cell activation. After TCR engagement, BTK was activated and subsequently activated PLCγ1, which amplified downstream TCR signaling and facilitated T-cell activation and expansion (70). Clinically, long-term treatment with ibrutinib, an inhibitor that forms irreversible covalent bonds to BTK, reversed CD8 T cell exhaustion and protected T cells from proliferation-induced senescence in chronic lymphocytic leukemia (B-CLL) patients. In addition, T cells from ibrutinib-treated CLL patients have decreased PD-1, TIM3, and LAG3 expression and increased antigen-specific responses (71–73). Ibrutinib improved CAR T cell expansion *in vitro* and promoted a less-differentiated less-exhausted naïve-like phenotype by inhibiting interleukin-2-inducible T-cell kinase (ITK) (31, 32). To date, several clinical trials are investigating the regimen of concurrent administration of CAR T cells and ibrutinib in B-cell malignancies (NCT02640209, NCT03960840). Concurrent ibrutinib therapy may improve CD19 CAR T-cell engraftment, enhance anti-tumor efficacy, and decrease CRS severity, leading to high rates of minimal residual disease (MRD)-negative responses, but progression-free survival (PFS) was unchanged (32–35). Second-generation BTKi acalanrutinib and zanubritinib, which are more selective and well-tolerated, have also been examined (74). Acalabrutinib improved CAR T-cell effector function and prolonged survival of tumor-bearing mice when combined with CAR T cells (36), while zanubrutinib lacked these positive effects (37).

#### Tyrosine kinase inhibitors (TKI)

Dasatinib, a second-generation tyrosine kinase inhibitor (TKI), was initially approved by FDA to treat Ph+ chronic myeloid leukemia (CML) (75). A recent study showed that dasatinib prevents or reverses CD28/CAR T and 4-1BB/CA T cell differentiation and exhaustion during *ex vivo* expansion, resulting in profoundly enhanced therapeutic efficacy and *in vivo* persistence (38). Multiple pathways are involved in this process, including Src phosphorylation, JAK/STAT, MAPK, and PI3K/AKT (38, 76).

### Epigenetic modulators

Epigenetic modulators represent another promising strategy to enhance T-cell function based on the epigenetic remodeling and chromatin transitions discovered during the process of T-cell exhaustion. DNA methyltransferases and histone deacetylases (HDACs) are activated during T-cell differentiation, resulting in high levels of DNA and histone methylation in exhausted T cells (7, 9). Recent studies revealed that decitabine, a clinical DNA methylation inhibitor, enhances anti-tumor activities, cytokine production, and CAR T cell proliferation in both *in vitro* and *in vivo* non-Hodgkin lymphoma (NHL) models (39). Decitabine also promotes the maintenance of effector function and the memory phenotype of NY-ESO-1-specific eTCR T cells leading to greater anti-AML efficacy (40). Likewise, HDAC inhibitors panobinostat, SAHA, and sulforaphane promote the generation of T cells with a central memory phenotype and reduce expression of immunosuppressive markers (PD-1, CTLA-4, TET2) in CAR T-cell, resulting in enhanced anti-tumor response in solid tumor models (41–43).

BRD4 is a member epigenetic modulator of the bromodomain and extra terminal motif (BET) subfamily. BRD4 promotes T_EM_ CD8 T-cell differentiation by regulating BATF expression. Treatment of CAR T cells with BET inhibitor JQ1 promoted the expansion of less differentiated T_SCM_ and T_CM,_ downregulated PD-1 and TET2 exhaustion marker expression, improved persistence and effector function, and augmented T-cell mediated anti-tumor effect in leukemia models (44, 45). Interestingly, the commonly used PI3K inhibitor LY294002 is also an inhibitor of BET bromodomains (77).

### Immune modulators

Immunomodulatory imide drugs (IMiDs) are thalidomide analogs with pleiotropic anti-myeloma properties. IMiDs act directly on malignant cells and indirectly *via* enhancing T and NK cell effector functions (78). Early *in vitro* studies showed that IMiDs induced T-cell proliferation, IL-2 and IFN-γ secretion, and myeloma-specific T-cell responses (46, 79). Myeloma patients treated with lenalidomide had increased numbers of central (T_CM_) and effector (T_EM_) memory CD8 T cells with decreased PD-1 expression (80). However, the effect of IMiDs on Treg remains uncertain (81). In general, favorable clinical outcomes with lenalidomide were observed from either induction or post-autologous stem cell transplant (ASCT) consolidation and maintenance (81). Aligned with previous findings, lenalidomide-treated CAR T cells acquired a memory phenotype, enhanced polyfunctional cytokine secretion, and increased immune synapse formation (47). CS1 CAR T cells expanded *in vitro* with lenalidomide had improved anti-tumor efficacy and *in vivo* persistence in murine myeloma models (47). Preclinical studies showed treatment with lenalidomide during the early phase of *in vivo* CAR T cell expansion recapitulated the effects of *ex vivo* lenalidomide exposure in multiple hematologic and solid tumor mouse models (48–52). As expected, several clinical trials investigating the combination of CAR T cells with lenalidomide have been initiated (NCT03070327, NCT05032820, NCT04923893, NCT04002401). Preliminary data has shown that early lenalidomide infusion enhances CAR T-cell response in patients with R/R DLBCL (53, 82).

## Implications of emerging peptide-based antagonists in ACT

### VIP-receptor antagonists (VIPR-ANT)

Vasoactive intestinal peptide (VIP) is a 28-amino acid neuropeptide isolated in 1970 from porcine duodenum that induces vasodilation and hypotension (83, 84). The immunosuppressive properties of VIP were described in the early 2000s by Delgado, who noted that VIP promoted the survival of Th2 effectors, the generation of memory Th2 cells, and enhanced Treg function (85–87). More recent studies showed that VIP enhances M2 macrophage polarization and promotes macrophages with a less-inflammatory physiologic profile that promotes tissue repair (88–90). We recently noted that VIP produced by activated T cells limits their proliferation *in vitro* (91), and VIP produced by donor plasmacytoid dendritic cells (pDCs) limits Graft-versus-Host Disease (GVHD) *in vivo* (92). The emerging immunoregulatory role of VIP on innate and adaptive immune functions makes it a candidate immunotherapy target.

VIP-hybrid (VIPhyb) is a VIP-receptor antagonist synthesized by replacing the six N-terminal residues of VIP with highly charged residues from the N-terminal peptide sequence of neurotensin (93). VIPhyb acts as a competitive antagonist, binding to VIP receptors VPAC1 and VPAC2 without activating the downstream VIP-receptor signaling pathway (94). In the past decade, our group showed that inhibiting VIP signaling could enhance CD8 T-cell proliferation and function, leading to favorable T-cell-dependent anti-viral and anti-cancer responses in murine models of CMV infection and acute leukemia, respectively (91, 95–98). To further improve the efficacy of VIP-receptor antagonists as immuno-modulatory drugs, we have developed a series of peptides, including ANT008, ANT308, and ANT195, that are predicted to have increased binding affinity to human VIP receptors VPAC1 and VPAC2 and have enhanced ability to elicit T cell-dependent antileukemia responses in mice (Li unpublished). Recently, we published that VIP-receptor antagonists (ANT008, ANT308) were synergistic when added to anti-PD1 antibodies in enhancing T-cell mediated anti-tumor response to multiple murine models of pancreatic ductal carcinoma (PDAC) (99). These exciting findings validated using VIP-receptor antagonists as anti-cancer immunotherapy agents. In addition, we further investigated the feasibility of using VIP-receptor antagonists in adoptive T-cell therapy.

### Synergy with PI3Ki to enhance T cell persistence and functionality

#### Idelalisib and VIPhyb

**Figure 1:**
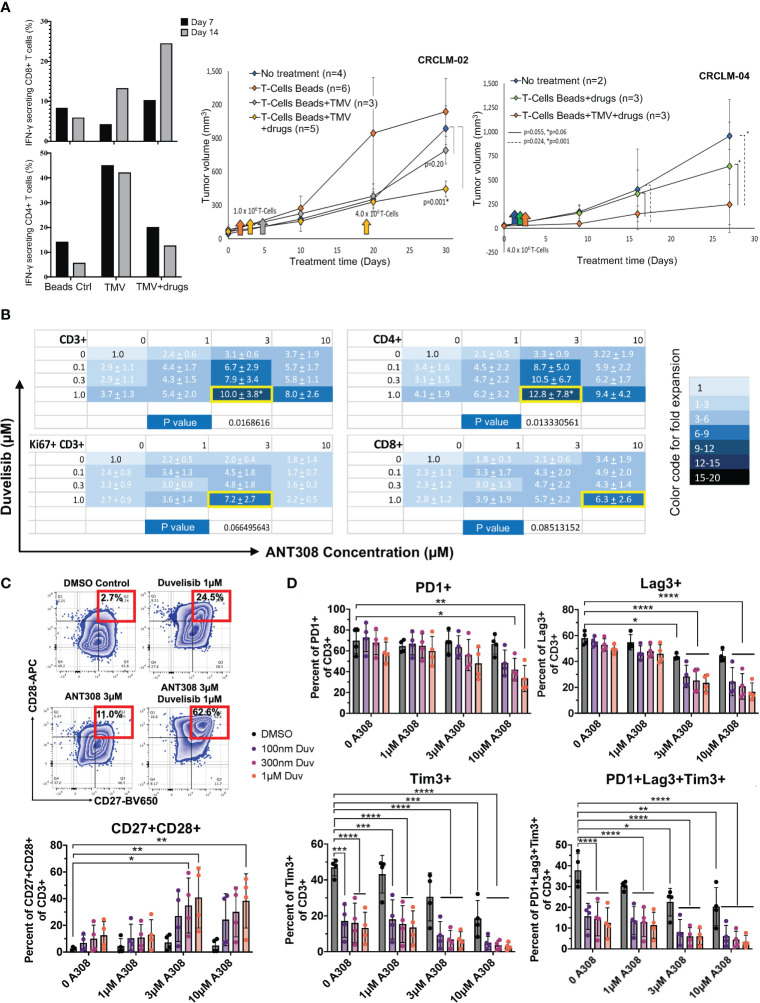
Pharmacological blockade of PI3K and VIPR signaling improves T-cell expansion and function *in vivo* (**(A)** adoptive antigen-specific T cells in metastatic colon cancer PDX model) and *in vitro* (**(B–D)**, human T cells). **(A)** Left: Increased IFN-γ secreting CD8+ T cells using decorated TMV with VIPhyb and idelalisib (CRCLM-02, n=1); Right: Decreased tumor growth in PDX (CRCLM-02 and CRCLM-04) mice receiving T cells expanded with beads+TMV+drugs. ANOVA was used to determine significance. The standard error (SE) was shown. **(B)** Total CD3+ T cells, the actively proliferating Ki67+CD3+ subset, CD4+CD3+ T cells, and CD8+CD3+ T cells are synergistically expanded *in vitro* by the combination of ANT308 and duvelisib. The mean +/- SD fold increase in cell expansion over control cultures containing neither added ANT308 nor duvelisib is shown (n=4), with color shading according to the relative increase. The pair of concentrations yielded the maximal increase in mean fold expansion is shown with a yellow border around the cell. **(C)** Frequencies of CD27+CD28+ T cells in cultures with duvelisib and ANT308 led to the highest average expansion for that subset of T cells (n=4). An example of gating is on the left. **(D)** ANT308 and duvelisib demonstrated synergy in decreasing PD1+, Lag3+, Tim3+, and PD1+Lag3+Tim3+ cells (n=4). Figures were plotted with Microsoft Excel and Prism 9. Paired two-sided student t-test was used to determine significance. *p<0.05, **p<0.01, ***p<0.001, ****p<0.0001.

Previously, our team has shown that idelalisib and VIPhyb synergically increased the transduction and expansion of anti-CD5 CAR T cells manufactured from DLBCL patients (21). The addition of idelalisib and VIPhyb to cultured T cells reduced terminal differentiation, enhanced cytokine expression, and preserved expression of costimulatory molecules CD27 and CD28 (21). These agents target distinct signaling pathways, and their combinatorial synergy might be applicable for manufacturing CAR T therapy in patients with hematological and in adoptive T cell for patients with solid tumor malignancies. However, applying adoptive T cell therapy to solid tumor patients is constrained by the low frequency of tumor-infiltrating lymphocytes and the high molecular heterogeneity of solid tumors lacking expression of public (shared) tumor antigens. Therefore, we tested the feasibility of expanding patient tumor-specific T cells *ex vivo* by adding idelalisib and VIPhyb, using matched tumor and PBMCs from consented metastatic colon cancer patients. The source of tumor antigens were tumor membrane vesicles (TMVs) manufactured from that patient’s tumor and decorated with IL-12 and B7-1 (100, 101). Activating TMV-stimulated autologous T cells in the presence of idelalisib, VIPhyb, and anti-CD3 Dyna beads expanded cancer-specific T cells (**Figure S1**). After 14 days of *ex vivo* culture, T cells expanded with decorated TMV, VIPhyb, and idelalisib had 25% of IFN gamma-expressing CD8 T cells compared to 13% IFN gamma-expressing CD8 T cells cultured with IL12/B7-1 decorated TMV without VIPhyb and idelalisib (**Figure 1A**). Notably, this effect was not observed in CD4 T cells, which may attribute to the lack of VPAC1 expression in CD4 subset (Passang unpublished). The tumor-antigen-stimulated T cells expanded with VIPhyb and idelalisib were more effective in controlling the growth of patient-derived colon cancer xenografts (PDX) following infusion into tumor-bearing immunodeficient NSG mice than T cells expanded with TMV but without the addition of VIPhyb + idelalisib, or T cells expanded with neither TMV nor VIPhyb plus idelalisib (**Figure 1A**).

#### Duvelisib and ANT308

Later we investigated the synergy effect of PI3Kγ/δ inhibitor duvelisib and leading VIPR-ANT peptide ANT308. The actively proliferating T cells (Ki67^+^CD3^+^), CD4+, and CD8+ T-cell subsets synergistically expanded *in vitro* with the combinatorial use of duvelisib and ANT308 (**Figure 1B**). We also found that ANT308 as a single agent, could promote a less senescent T-cell phenotype (CD27^+^CD28^+^), decrease exhausted T cells (PD1^+^Lag3^+^Tim3^+^) and reduce the expression of PD-1, LAG3, and TIM3. Adding duvelisib to T cells cultured with ANT308 or ANT195 synergistically enhanced the expansion of central memory T cells. The percentage of T cells co-expressing CD27 and CD28 increased from 2.59% to 7.16% with single-agent ANT308 (3 μM) and 12.5% with single-agent duvelisib (1 µM). The percentage of CD27+CD28+ T cells further increased to 40.83% with the combination of ANT308 and duvelisib (**Figure 1C**). Similarly, the percentage of T cells with an exhausted phenotype (PD1^+^Lag3^+^Tim3^+^) decreased from 37.8% in control cultures with neither ANT308 nor duvelisib, to 22.53% with only ANT308, to 12.56% with single-agent Duvelisib. Adding both drugs together further decreased the frequency of exhausted T cells to 5.93% (**Figure 1D**). Comparable synergistic effects of adding the VIP-receptor antagonist ANT195 to duvelisib effect were observed (**Figures S2-3**).

## Concluding remarks

The field of cell-based immunotherapy is growing exponentially. However, efforts are still needed to improve the clinical response rate. Recent studies have focused on developing strategies to optimize efficiency in manufacturing T-cells for ACT therapy and the efficacy of T cells *in vivo*. We have reviewed current strategies to enhance T-cell expansion, persistence, and functionality during *ex vivo* manufacturing. Moreover, we further discussed the synergistic benefits of approaches that target multiple signaling pathways. Besides the conventional small-molecule drugs, novel VIPR-ANT peptides are promising immunotherapeutic candidates. Future studies will define the immunoregulatory role of VIP in ACT and its feasibility in clinical application.

## Data availability statement

The raw data supporting the conclusions of this article will be made available by the authors, without undue reservation.

## Ethics statement

The animal study was reviewed and approved by Emory University Institutional Animal Care and Use Committee.

## Author contributions

HZ conceived, drafted, and wrote the manuscript. TP performed the *in vitro* experiments and provided the data of T-cells treated with Duvelisib and ANT308. SR led the adoptive T cell therapy project in metastatic colon cancer and conducted *in vivo* PDX-model experiments. RB and PS produced TMV and incorporated the molecules by protein transfer. MJ and LY established the PDX model. CP critically reviewed and revised the manuscript. EW edited and revised the manuscript and provided funding support. All authors contributed to the article and approved the submitted version.
