# Supplementary material for: The magic of small-molecule drugs during ex vivo expansion in adoptive cell therapy
Source: Front Immunol. 2023 Apr 21;14:1154566. doi: 10.3389/fimmu.2023.1154566 (PMC10160370; doi:10.3389/fimmu.2023.1154566)
Supplement: Supplementary file 1 [file DataSheet_1.docx]

**Supplementary Figure 1**


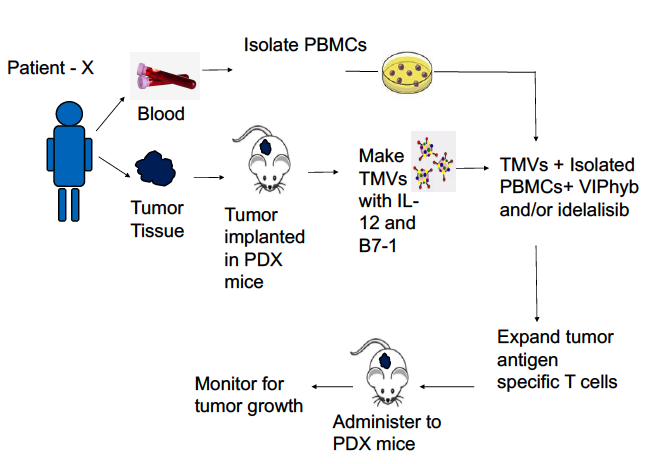


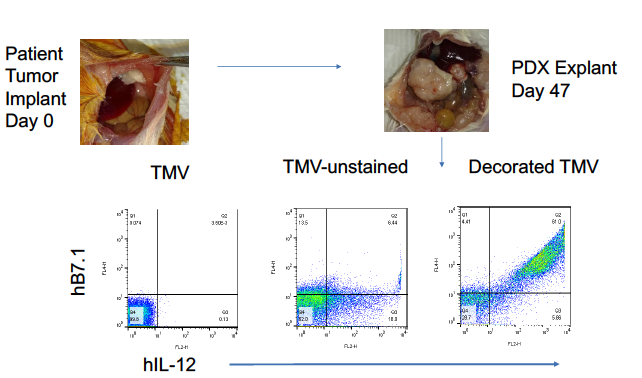


**Figure S1. (Top) Approach to expand tumor-specific T cells with enhanced anti-tumor activity.** Tumor tissue acquired from the 1^st^ generation PDX mice was used to make tumor membrane vesicles (TMVs) decorated with IL-12 and B7-1. Antigen-specific T cells were obtained by expanding isolated T cells with decorated TMVs. **(Bottom) PDX models of human colon cancer and decorated TMVs.**

**Supplementary Figure 2**

Color code for fold expansion

**ANT195 Concentration (µM)**

**Duvelisib (µM)**


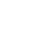

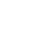

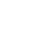

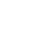


**Figure S2. Synergistic effect of Duvelisib and ANT195.** Total CD3+ T cells, the actively proliferating Ki67+CD3+ subset, CD4+CD3+ T cells, and CD8+CD3+ T cells are synergistically expanded in *vitro* by the combination of ANT195 and duvelisib (n=4). The mean +/- SD fold increase in cell expansion over control cultures containing neither added ANT195 nor duvelisib is shown, with color shading according to the relative increase. The pair of concentrations yielded the maximal increase in mean fold expansion is shown with a yellow border around the cell. Paired two-sided student t-test was used to determine significance. **p<0.05, **p<0.01, ***p<0.001, ****p<0.0001*

**Supplementary Figure 3**

**Lag3+**

**PD1+**

**Figure S3. Synergistic effect of Duvelisib and ANT195.** ANT195 and duvelisib demonstrated synergy in decreasing PD1+, Lag3+, Tim3+, and PD1+Lag3+Tim3+ cells (n=4). Figures were plotted with Microsoft Excel and Prism 9. Paired two-sided student t-test was used to determine significance. **p<0.05, **p<0.01, ***p<0.001, ****p<0.0001*

**PD1+Lag3+Tim3+**

**Tim3+**

**Methods**

**Patient samples**

Peripheral blood mononuclear cells (PBMCs) were obtained from consenting healthy donors and patients with metastatic colon cancer via apheresis or phlebotomy and SepMate^TM^ (STEMCELL Technologies, Vancouver, Canada) separation of peripheral blood. All samples were processed within 24 hours after collection, frozen in Cryostor CS10 (STEMCELL Technologies), and stored in liquid nitrogen until further use. Paired patient tumor samples were obtained from consenting patients. Tumor samples were processed immediately and implanted in NSG mice from The Jackson Laboratory. Tumor membrane vesicles (TMV) were prepared using patient tumors (Isoplexis Corporation, CT) and later decorated with GPI-anchored IL-12.

**PDX model**

Six- to 8-week-old male NSG mice (NOD.Cg-*Prkdc^scid^ Il2rg^tm1Wjl^*/SzJ) were purchased from The Jackson Laboratory. Metastatic colon cancer PDX models were established with the support of Dr. Lily Yang’s lab from surgically resected colon cancer of two patients (CRCLM-02 and CRCLM-04). Small fragments (1-2 mm) of fresh cancer tissues were implanted into the mammary fat pad of NSG mice using a surgical procedure. Orthotopic PDX tumors grew for 47 days. PDX tumors were removed after the mice were sacrificed. Fresh tumor fragments were implanted into the mammary fat pad of NSG mice for adoptive T-cell experiments. All procedures were approved by the Emory University Institutional Animal Care and Use Committee and conformed to the “Guide for the Care and Use of Laboratory Animals.”

**Compounds**

Idelalisib (CAL-101) was purchased from BocSci (Shirley, NY) and stored as a 10 mM stock solution in dimethyl sulfoxide (DMSO) at -20°C. Duvelisib was purchased from SelleckChem (Houston, TX) and stored as a 20 mM stock solution in DMSO at -20°C. VIPhyb (KPRRPYTDNYRELRKQMAVKKYLNSILN) was purchased from New England Peptide (Gardner, MA) and reconstituted in sterile molecular-grade water. VIP308 and VIP195 are modified peptide sequences of VIPhyb. The peptides were purchased from RS synthesis (Louisville, KY) at a purity of >95% and reconstituted in sterile molecular-grade water.

***Ex vivo* T cell expansion**

T cells were isolated from PBMCs of 4 healthy donors using a pan-T cell isolation kit from Miltenyi Biotec (Catalog no. 130-096-535). Purified T cells were then cultured in complete RPMI in the presence of 30IU/ml IL2 for 7 days. T cells were stimulated with CD3/CD28 beads at 1:1 cells to beads ratio on day 0. Cells were treated either with the addition of ANT308 or ANT195 alone (0, 1, 3 or 10 uM) or in combination with Duvelisib (0, 0.1, 0.3, or 1 uM). Duvelisib was added to media on day 0 and day 3, whereas ANT308 or ANT195 were added daily. At day 7, CD3/CD28 beads were removed via handheld 96-well magnetic separator and cells were collected for flow cytometric analysis and stained with directly conjugated monoclonal antibodies to CD3, CD4, CD8, CD27, CD28, Tim3, Lag3, and Ki67 (Biolegend, CA). T cells were counted via volumetric counting by flow cytometry as measured as counts/ul. The mean +/- SE fold increase in cell expansion over control cultures containing neither added VIP-R antagonist peptide nor Duvelisib is shown, with color shading according to the relative increase level. A paired two-sided student t-test compared the numbers of T cell/ul or percentage in the culture at 7 days at this specific combination of VIP-R antagonist peptide and Duvelisib in corresponding control cultures.
